# Barriers to Improving Pain Management in the Emergency Department: Lessons from a Lean-Driven Quality Improvement Initiative

**DOI:** 10.3390/jcm14134566

**Published:** 2025-06-27

**Authors:** Jakub Nożewski, Radosław Bondarczuk, Grzegorz Hołody, Meabh Kitt, Natalia Mućka, Urszula Religioni

**Affiliations:** 1 Department of Emergency Medicine, Dr Jan Biziel’s University Hospital No. 2, 85-168 Bydgoszcz, Poland; 2 Lean Medic Foundation, Ul. Władysława Łokietka 24, 05-230 Kobyłka, Polandgrzegorz.holody@leanmedic.pl (G.H.); 3 University Hospital Galway, H91 YR71 Galway, Ireland; meabhkitt20@gmail.com; 4 Collegium Medicum, Nicolaus Copernicus University, 85-094 Bydgoszcz, Poland; 5 School of Public Health, Centre of Postgraduate Medical Education, 01-826 Warsaw, Poland

**Keywords:** pain measurement, emergency medical services, oligoanalgesia, quality improvement, analgesia administration

## Abstract

**Background/Objectives**: Pain remains as a prevailing cause, prompting patients to seek medical attention, comprising approximately 40% of all emergency department (ED) visits annually. Timely and effective pain management is crucial for patient comfort, satisfaction, and optimal recovery. However, there is increasing evidence highlighting the concern that patients often receive inadequate pain management in both emergency departments and prehospital settings. Despite the simplicity and potential for the repetitive use of pain scales throughout a patient’s stay, it appears that a greater emphasis is often placed on monitoring hypotension or low saturation values rather than addressing pain levels above 7 on the numeric rating pain scale. **Methods**: This article represents an ambitious attempt to implement process improvement methodologies such as Lean Management and SixSigma, both which have been well established in service and industrial fields, within the hospital environment to improve the process of pain management in the emergency department. **Results**: The implementation of pain management improvement processes in the emergency department led to a statistically significant but clinically modest increase in the administration of analgesics and improved pain reporting practices. The percentage of patients receiving no analgesia decreased from 96.6% to 94.8% (*p* = 0.008), and the documentation of pain characteristics during triage improved. However, the escalation of pain therapy remained limited, and strong analgesics were still underutilized. **Conclusions**: Despite partial improvements, the lean management-based interventions did not sufficiently address the problem of oligoanalgesia in the emergency setting. Sustainable change requires enhanced clinical engagement, ongoing staff training, and the broader adoption of structured analgesia protocols across prehospital and hospital care.

## 1. Introduction

In-hospital medical care plays a pivotal role in providing prompt and effective assistance to patients during health emergencies. Pain is one of the most common symptoms reported by patients, and it can manifest with diverse causes and varying levels of intensity [1,2,3,4,5,6,7]. This includes both acute and chronic pain, which often coexist in the ED environment. Many patients with chronic conditions, such as breakthrough cancer pain or chronic low back pain, present to the ED due to limited access to outpatient care or delays in obtaining prescriptions. In ED settings, acute pain is a prevalent issue, but there are significant obstacles that impede the achievement of ideal pain management [8,9,10,11]. These obstacles include the insufficient use of pain-relieving medications and delays in administering them. Inadequately treated pain has detrimental effects on all aspects of the quality of patients’ life. Ineffective or delayed treatment of pain can lead to serious consequences, such as prolonged suffering, increased risk of cardiovascular complications, prolonged wound healing and even death [12,13,14,15,16]. Furthermore, insufficiently treated acute pain, and especially chronic pain, can lead to exclusion from professional, social, and family life. Such emotional distress even may contribute to depression, alcoholism, and suicide. The impact of pain can be seen in the global economy, where every year, the global economy loses trillions of dollars due to people suffering from pain and their subsequent absenteeism from work [17]. Based on an analysis of the American Pain Society, in 2012, healthcare expenditures related to pain in the US alone ranged from USD 560 to 635 billion [18]. Japan’s economy loses JPY 8.3 billion per year in lost productivity just because of chronic low back pain [19]. Although pain management is a fundamental human right [20,21], undertreated pain is not only related to acute trauma patients in overcrowded EDs or during acute prehospital situations. It also profoundly affects cancer patients, and despite the fact that almost 90% of cancer patients ideally should be pain free, many still suffer from undertreated pain [22]. Several factors contribute to the inadequate management of pain in emergency departments and prehospital settings. One significant challenge is that pain is invisible and subjective. Healthcare providers often rely on patients’ self-reported pain scores, which are based on their subjective perceptions of pain intensity. Rarely, it is considered as a fifth vital sign. Despite efforts to incorporate pain as a vital sign, its subjective nature remains a hurdle in providing consistent and effective pain management. Studies have shown that prehospital triage personnel and emergency department staff may sometimes underestimate the pain reported by patients [23]. This underestimation can occur due to various factors, such as healthcare providers relying on their own experiences and observations to assess pain levels rather than fully considering the patient’s subjective reporting [24,25]. It is important to treat pain like other measurable parameters, such as low blood saturation values, because, like them, pain is a critical indicator of the patient’s condition and should not be ignored solely on the basis of the patient’s external appearance or symptoms. After all, it seems inconceivable that a patient with a saturation value of 60% without obvious symptoms of shortness of breath, which we have seen many times during the covid pandemic, should not be given oxygen because they look healthy. In a comprehensive review, underestimation of pain by professionals compared to patients was reported in nearly 80% of studies [26]. In effect, even when the system works correctly, the patient may be directed to an ineffective pain management pathway. An issue related to healthcare providers is also the lack of current knowledge and training in pain management [27,28,29].

Many healthcare professionals practice outdated guidelines and are not familiar with the latest evidence-based guidelines for pain management [23,30]. This aspect is also interconnected to another factor that contributes to inadequate pain management—apprehension of drug-adverse effects or opioid dependency [31,32]. There are also systemic barriers to pain management in EDs and prehospital settings. For example, overcrowding and understaffing can make it difficult for healthcare providers to devote sufficient time and attention to managing pain in individual patients. Furthermore, EDs and prehospital settings may not have adequate resources, such as pain management specialists or pharmacists, to support healthcare providers in managing pain effectively [1,10,33].

## 2. Materials and Methods

### 2.1. Study Design and Setting

This was a prospective, single-center quality improvement study conducted in the emergency department of a large academic hospital in Poland. The study began with a one-week pilot phase from March 1 to 8 March 2023, during which all adult patients presenting to the ED were screened for eligibility. This phase served as the baseline diagnostic assessment of existing pain management practices and system inefficiencies.

Based on the findings from the pilot, the study team attempted to implement a structured improvement intervention using Lean Management principles and the Kocher model of healthcare transformation. Due to institutional constraints and limited uptake, the intervention was only partially implemented.

To monitor changes over time and assess the potential impact of both formal and informal process shifts, we conducted a prospective time series trend analysis. This included 9362 triage records collected over five consecutive three-month periods from March 2023 to May 2024. These records were automatically extracted from the ED’s electronic triage system and analyzed to evaluate trends in pain documentation and analgesia administration.

No retrospective data collection was performed at any stage of the study.

The primary outcome of the study was the proportion of patients with pain who received any analgesia during their ED stay. Secondary outcomes included the use of strong analgesics, changes in treatment rates across time and patient subgroups, quality of pain documentation, and administration of analgesia during early stages of care, particularly at triage.

### 2.2. Study Population

All adult patients presenting to the ED during the week of 1–8 March 2023 were screened for eligibility. Patients were included if they presented with pain as the chief complaint and provided informed consent. Exclusion criteria included the inability to consent, clinical instability requiring immediate life-saving intervention (e.g., STEMI), or the presentation for reasons unrelated to pain.

Of the 871 patients assessed, 232 met the inclusion criteria. After the exclusion of 14 records due to missing or ambiguous data, 218 patients were included in the final analysis. For the time series trend analysis, a separate cohort of 9362 triage records was analyzed over five three-month periods spanning March 2023 to June 2024.

### 2.3. Intervention

A multi-phase Lean intervention was deployed, beginning with process mapping workshops that included frontline ED staff, EMS personnel, nursing leadership, medical students, and Lean consultants. Based on the identified inefficiencies, targeted actions were implemented, including:

- Establishment of triage-area ampoule kits with analgesics
- Extension of medication authorization to ED nursing staff under protocol
- Standardized pain assessment protocols
- Internal educational campaigns and visual materials
- Implementation of pain-related documentation fields in the electronic medical record

All interventions were prioritized using a feasibility–impact matrix and aligned with the DMAIC (Define, Measure, Analyze, Improve, and Control) cycle. The initial Define phase involved collaborative engagement with the Emergency Department (ED) team and management to confirm the process scope and problem definition. A high-level process map was developed to identify potential intervention points for pain management within the existing clinical workflow.

During the Measure phase, a structured data collection methodology was designed. Standardized data collection forms were developed and subsequently utilized by ED paramedics and nurses to prospectively gather relevant data.

The Analyze phase employed rigorous statistical analysis of the collected data. Root cause analysis techniques, including Ishikawa diagrams and the 5 Whys methodology, were applied to elucidate the underlying factors contributing to inadequate pain management. Brainstorming sessions and cost-effectiveness analysis informed the identification of potential improvement strategies during the subsequent Improve phase.

Finally, the Control phase focused on establishing robust monitoring mechanisms. Key performance indicators (KPIs) and relevant metrics were defined to enable ED managers to prospectively track and ensure the sustained efficacy of pain treatment protocols.

### 2.4. Data Collection

Pain scores, medication administration events, and treatment timelines were recorded using a structured form accompanying the patient throughout their ED stay. Data were collected at five key points: prehospital care (EMS), triage, initial physician evaluation, waiting period post-consultation, and final disposition. In parallel, triage data from over 9000 patient records were retrospectively analyzed to assess pain trends and treatment uptake over time.

Patient pain intensity was recorded using the standard 0–10 numeric rating scale (NRS). Additional variables collected included age, sex, pain localization, etiology (traumatic/non-traumatic/chronic), treatment refusal and its reason, and drug type administered. In addition to the prospective patient cohort, we retrospectively analyzed 9362 triage records covering the full timeline of the intervention. These were divided into five consecutive three-month periods to assess pre- and post-intervention trends in analgesia use and documentation quality. The period definitions are presented in Table 1.

Period 2 was designated as the primary comparator, due to consistent documentation quality and organizational stability at that stage.

### 2.5. Outcome Measures

The primary outcome was the proportion of patients with pain who received any analgesia during their ED stay.

Secondary outcomes included:

- Proportion of patients receiving strong analgesics (e.g., morphine and fentanyl)
- Change in analgesia administration rates over five time periods
- Variation in treatment rates across age groups and ED care stages
- Documentation of pain characteristics and chronicity
- Reasons for non-treatment or patient refusal

### 2.6. Statistical Analysis

Descriptive statistics were used to characterize the study population. Categorical variables were presented as frequencies and percentages. Comparative analyses between time periods and patient groups were performed using chi-square tests or Fisher’s exact tests when appropriate. A significance level of *p* < 0.05 was used for all inferential testing. The statistical analysis was conducted using R software (version 4.4.2).

### 2.7. Ethical Considerations

The study was approved by the local Bioethics Committee. Participation in the prospective phase was voluntary, and written informed consent was obtained from all patients. The study was classified as a quality improvement initiative, and no experimental treatments were introduced. All interventions adhered to institutional and national guidelines on pain management.

## 3. Results

### 3.1. Patient Profile and Pain Characteristics

**Figure 1:**
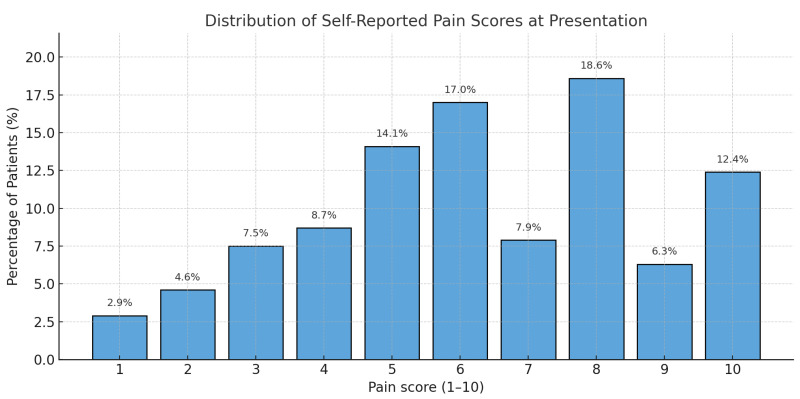
Distribution of self-reported pain scores at presentation (NRS 0–10 scale).

Out of 871 ED presentations screened, 218 patients met the inclusion criteria for pain as the chief complaint. Female patients were slightly more represented (56.4%). The majority (76.5%) reported moderate to severe pain (scores ≥ 5/10). Pain etiology was predominantly non-traumatic (60%), followed by traumatic causes (38.5%) and exacerbations of chronic pain (1.5%). Although detailed coding of pain etiology was not performed, clinical documentation suggests that the most common non-traumatic pain presentations included renal colic, abdominal pain of unknown origin, and lower back pain. Within the traumatic pain group, ankle sprains and wrist injuries were predominant. Pain distribution patterns indicated occasional values of 0–1, likely reflecting inadequate patient understanding of pain scales (Figure 1).

### 3.2. Drug Administration Patterns Across Stages

**Figure 2:**
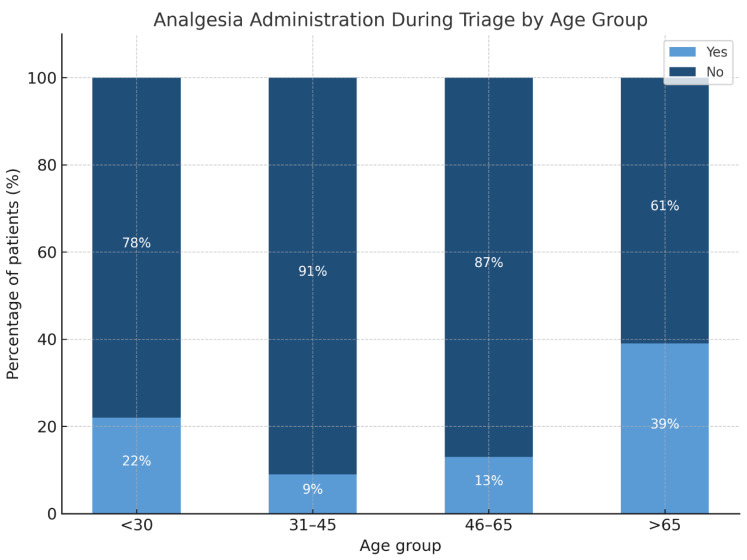
Proportion of patients receiving analgesia during triage, stratified by age group.

Among the five defined stages of the patient journey, 83% of all analgesic administrations occurred during three phases: physician examination (45%), triage (23%), and prehospital care by EMS (15%). Although no major age-related differences were observed overall, a significant disparity emerged during the triage phase: patients aged 31–45 had the lowest rate of analgesia (9%), whereas those >65 had the highest (39%) (Figure 2).

**Figure 3:**
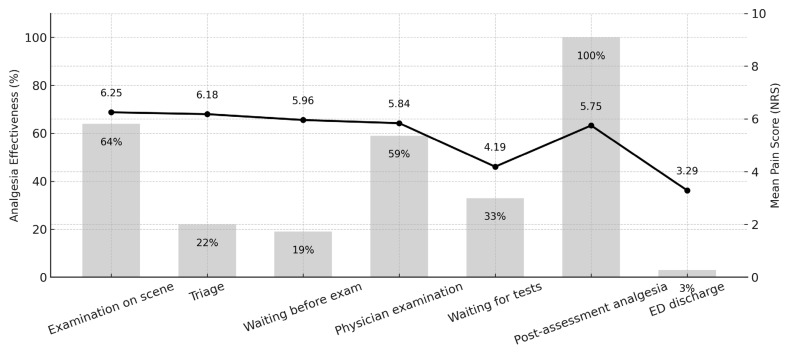
Analgesia effectiveness and mean pain intensity across emergency department care stages. Bar graph represents the proportion of patients receiving analgesia; the line represents mean pain scores on the NRS scale.

Analgesia effectiveness and mean reported pain intensity across all stages of care are shown in Figure 3. The post-assessment phase—during which nurses initiated treatment based on condition reassessment—showed the highest rate of treatment (100%), though the mean pain scores remained moderate (NRS 5.75). In contrast, both waiting periods (pre- and post-examination) were associated with low treatment rates and only gradual reductions in pain scores.

### 3.3. Medication Use and Shifts over Time

**Figure 4:**
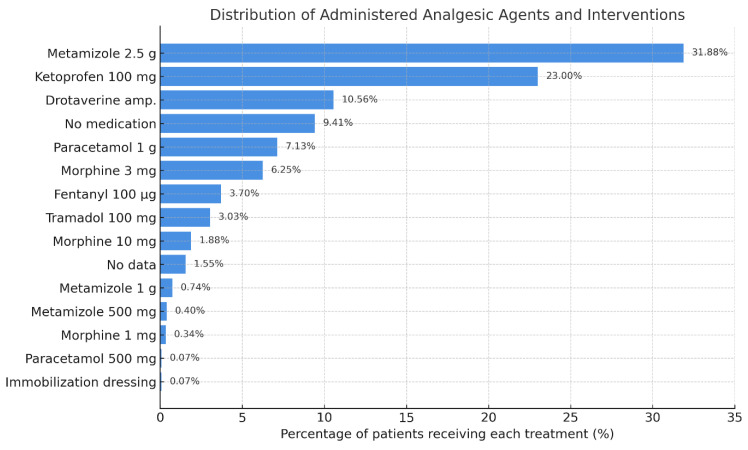
Distribution of administered analgesic agents and related interventions in the ED cohort. Bars represent the percentage of patients receiving each agent. Data include both pharmacologic treatments and supportive measures.

Within the hospital, metamizole sodium (2.5 g) was the most commonly administered analgesic (31.9%), followed by ketoprofen (100 mg; 23%) and drotaverine hydrochloride (10.5%). Opioid use remained limited: morphine (combined doses from 1 to 10 mg) was administered in only 8.5% of patients (Figure 4).

In the prehospital setting, analgesic administration by EMS was rare (5.96% overall), although a statistically significant upward trend was observed (from 3.4% to 5.2%; *p* = 0.008) between Period 2 and Period 5 (Table 2). Notably, fentanyl and pyralgina showed a modest but measurable increase in use over time.

### 3.4. Treatment Gaps and Patient Refusal

Across all stages, the proportion of patients who received no analgesia decreased modestly but significantly, from 96.6% (Period 2) to 94.8% (Period 5) (*p* = 0.008). Despite this, under-treatment persisted across both EMS and ED settings.

The most frequently documented reason for non-treatment was patient refusal (59%), followed by recent self-medication at home (13%) and perception of tolerable pain (8%).

### 3.5. Root Cause Analysis

A structured workshop with frontline staff led to the identification of three primary domains contributing to oligoanalgesia:

1. **Human Factors**—Limited staff engagement and knowledge gaps.
2. **Environmental Constraints**—Fear of masking symptoms, medicolegal concerns, and institutional inertia.
3. **Procedural Barriers**—Lack of formal authorization for non-physician drug administration during early phases.

**Figure 5:**
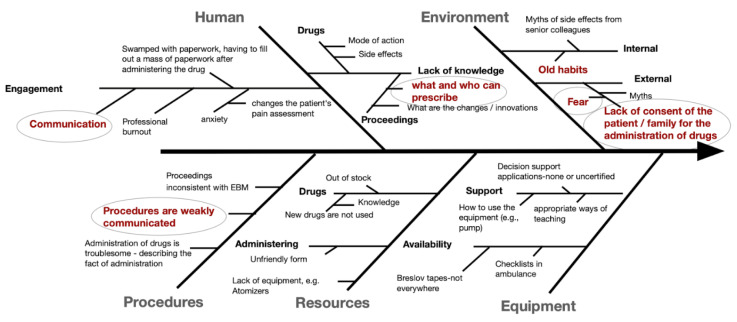
Ichikawa (fishbone) diagram illustrating the contributing factors to inadequate pain management in the emergency department.

These were summarized via a fishbone diagram, facilitating the prioritization of interventions (Figure 5).

### 3.6. Improvement Strategy and Implementation Targets

**Figure 6:**
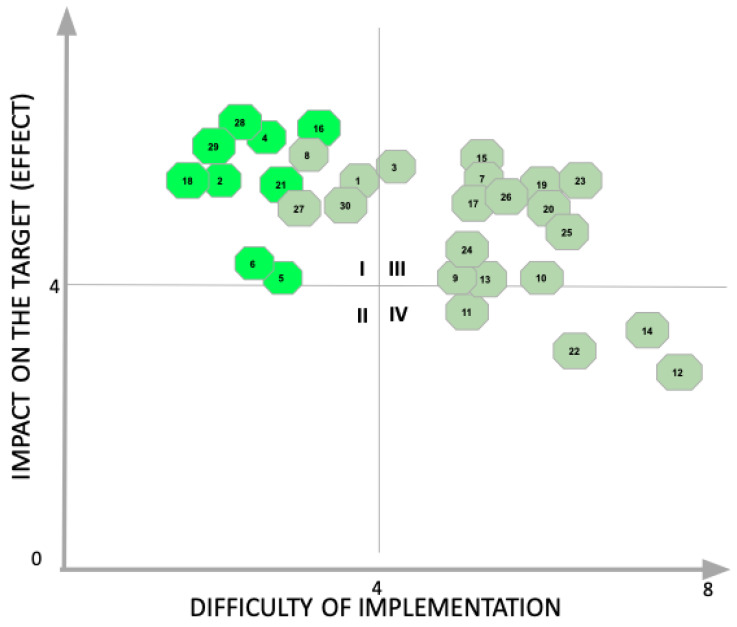
Impact–difficulty matrix used to prioritize improvement actions targeting pain management in the emergency department. Interventions in the top-left quadrant were selected for implementation based on high expected impact and low implementation complexity.

Following analysis, an action matrix was developed based on impact and feasibility. Eight interventions were selected for implementation, including triage ampoule kits, expanded ED staff prescribing rights, patient education tools, and pain documentation protocols (Figure 6 and Table S1—Supplementary Materials).

## 4. Discussion

While the implementation of early pain management protocols in emergency departments (EDs) is widely supported in the literature for improving patient satisfaction, operational efficiency, and clinical outcomes, our results demonstrate that even well-structured interventions may yield only marginal benefits if broader systemic and cultural factors are not addressed [34,35].

A modest but statistically significant decrease was noted in the proportion of patients receiving no analgesia (from 96.6% to 94.8%, *p* = 0.008). Moreover, there was a slight increase in the administration of both first-step analgesics (e.g., metamizole) and strong opioids (e.g., fentanyl). Triage-based pain assessment practices also showed measurable improvement. However, these gains fell short of our initial expectations. Prior to conducting the statistical analysis, the visible process improvements and staff engagement had suggested more substantial progress. This divergence between perceived and actual impact underscores the importance of rigorous outcome evaluation and continuous monitoring in quality improvement initiatives.

Our findings align with the broader literature emphasizing the complexity of ED pain management. The Pain in Emergency Medicine Initiative (PEMI) by Todd et al. demonstrated that even multicenter, standardized interventions struggled to overcome entrenched barriers to effective analgesia [36]. More recently, Sadasivam et al. showed that door-to-analgesia time could be dramatically reduced—from 55.5 min to 15 min—through focused PDSA cycle interventions targeting musculoskeletal injuries [37]. While our framework shared many methodological components—process mapping, standardized assessments, staff education, and feedback—our broader, undifferentiated patient scope may have diluted the measurable impact of any single change.

Notably, our intervention spanned all ED patients presenting with pain, rather than targeting a specific clinical subgroup. This comprehensive design was intentional, reflecting the widespread nature of oligoanalgesia across emergency care, but it likely made rapid gains more difficult to achieve. Our findings reinforce a central insight from both the PEMI and Sadasivam studies: successful improvement efforts require not only structured tools but deep contextual sensitivity and cultural readiness. While we collected NRS scores for all patients, analgesic selection was more closely aligned with pain etiology than numeric intensity alone. National recommendations emphasize tailoring treatment to the clinical context, e.g., using non-opioids for severe renal colic or muscle pain, even when NRS ≥ 8.

This observation is further supported by a 2024 review by Nagpal et al. [38], which emphasized that pain management in EDs continues to suffer from inconsistency, despite the widespread availability of effective analgesic options and guidelines. The authors identified key structural and behavioral barriers, including time constraints, poor adherence to protocols, inadequate staff training, and a lack of multimodal approaches. They concluded that “standardized pain assessment tools and continuous staff education remain underutilized across most EDs” and called for the integration of tailored, multimodal strategies that align with real-world emergency workflows.

Similarly, Mostafa et al. [39] highlighted early pain assessment, structured treatment protocols, and Lean-based process optimization as essential for improving ED performance. They identified delays in analgesia as a key metric of system inefficiency and recommended real-time feedback mechanisms and adaptive staffing models as important enablers of pain management reform.

In our setting, persistent barriers—communication gaps, documentation lapses, fear of masking critical diagnoses, and medicolegal concerns—mirror the challenges described in these reviews. Campaigns such as Choosing Wisely have rightly argued that, without cultural change, even well-designed protocols will fall short [40,41,42]. Similarly, Morrison et al. (2006) found that education, audit feedback, and computerized decision support failed to reduce pain when not accompanied by behavioral and organizational reform [43,44]. These systemic challenges are further complicated by the increasing volume and complexity of ED visits, which strain resources and delay timely interventions. Patient populations such as older adults, people with dementia, or those in long-term care facilities may experience agitation that is misinterpreted as delirium or infection rather than unrecognized pain. This diagnostic overshadowing, especially in non-communicative or cognitively impaired patients, has been documented as a major cause of oligoanalgesia in both prehospital and ED settings [11]. Addressing these subtler barriers requires not only protocol adherence but a shift in provider awareness and diagnostic sensitivity. In contrast, participatory approaches such as Minaya-Freire’s work on dementia patients (2020) demonstrate how co-designed, staff-driven solutions tailored to local needs may be more effective [45]. Another relevant limitation is that our study did not address the use of locoregional anesthesia techniques, which are increasingly recognized as effective tools for targeted pain control. However, such interventions are not routinely performed at the triage stage in our setting and are currently beyond the scope of practice for nurses and paramedics under Polish regulations. As our quality improvement initiative focused on early-stage, protocolized analgesia—often initiated before physician involvement—our analysis was limited to pharmacologic methods that can be administered by non-physician personnel. Additionally, the study has several other important limitations. The prospective data collection was limited to a single week, which may not capture seasonal or operational variability. No control group or randomization was included, which restricts causal inference. Furthermore, our analysis relied on the accuracy of ED documentation, which was incomplete in many cases, particularly regarding reasons for treatment refusal. Finally, we focused on process indicators rather than clinical outcomes such as actual pain reduction or patient satisfaction, which limits the assessment of real-world therapeutic effectiveness.

Taken together, these findings suggest that Lean and QI methodologies can offer a powerful structure for identifying bottlenecks and launching interventions but only when integrated into a broader, adaptive system of change. Pain management is not just a clinical task; it is a cultural and organizational process, dependent on alignment across practice patterns, institutional priorities, and patient trust.

## 5. Conclusions

The implementation of Lean-based process improvement strategies in the emergency department led to a modest but statistically significant increase in analgesic use and improved documentation of pain assessment, particularly during triage and prehospital care. Despite high levels of staff engagement and clear workflow improvements, oligoanalgesia remained prevalent, underscoring the limitations of operational changes when not embedded within broader clinical and cultural reforms. As highlighted in the recent literature, sustainable improvement in ED pain management requires a combination of process redesign, continuous feedback, and staff empowerment. Our findings suggest that Lean methodologies offer valuable structure for initiating change but must be integrated into context-sensitive, multidimensional strategies to achieve lasting impact.

## Figures and Tables

**Table 1:** Time period definitions used for the trend analysis of triage records.

Period	Time Range	Purpose in Analysis
Period 1	Mar 2023–May 2023	Pre-implementation/pilot
Period 2	Jun 2023–Aug 2023	Baseline comparison (pre-intervention)
Period 3	Sep 2023–Nov 2023	Early intervention phase
Period 4	Dec 2023–Feb 2024	Mid-phase implementation
Period 5	Mar 2024–Jun 2024	Post-intervention follow-up

**Table 2:** Prehospital analgesic administration by emergency medical services across defined time periods.

Analgesic	Period 1 (N = 1436)	Period 2 (N = 1646)	Period 3 (N = 1884)	Period 4 (N = 1871)	Period 5 (N = 2525)	Total (N = 9362)	*p*-Value
No medication	1137 (93.11%)	1590 (96.6%)	1741 (92.41%)	1742 (93.11%)	2394 (94.81%)	8804 (94.04%)	* 0.008
Fentanyl 100 µg	21 (1.46%)	11 (0.67%)	23 (1.22%)	19 (1.02%)	22 (0.87%)	96 (1.03%)	
Ketoprofen 100 mg	18 (1.25%)	13 (0.79%)	20 (1.06%)	24 (1.28%)	18 (0.71%)	93 (0.99%)	
Morphine 1 mg	4 (0.28%)	2 (0.12%)	5 (0.27%)	2 (0.11%)	2 (0.08%)	15 (0.16%)	

* There was a statistically significant difference between the groups (*p* < 0.05).

## Data Availability

The data supporting the findings of this study are not publicly available due to institutional and legal restrictions on sharing identifiable patient health information. Reasonable requests for access to de-identified data may be considered by the corresponding author, subject to approval by the Ethics Committee of the University Hospital no. 2 in Bydgoszcz.

## Supplementary Materials

The following supporting information can be downloaded at https://www.mdpi.com/article/10.3390/jcm14134566/s1, Table S1. Improvement opportunities identified during the Lean workshop on emergency department pain management.

## Abbreviations

The following abbreviations are used in this manuscript:

DMAIC	Define, Measure, Analyze, Improve, Control
ED	Emergency Department
ED-TV	Emergency Department television
EMS	Emergency Medical Services
NRS	Numeric Rating Scale
PEMI	Pain in Emergency Department Initiative
QI	Quality Improvement
