# Supplementary material for: Barriers to Improving Pain Management in the Emergency Department: Lessons from a Lean-Driven Quality Improvement Initiative
Source: J Clin Med. 2025 Jun 27;14(13):4566. doi: 10.3390/jcm14134566 (PMC12249701; doi:10.3390/jcm14134566)
Supplement: Supplementary file 1 [file jcm-14-04566-s001.zip › jcm-3678897-supplementary.pdf]

Table S1. Improvement opportunities identified during the Lean workshop on emergency department pain management.

No.	Action	No.	Action
1	**Review of treatment instructions for applicability**	16	**Education on ED-TV**
2	**Pressure on Emergency Medical Services Team**	17	**BTS**—Base Transceiver Station: a wireless communication node installed in the ED to enable targeted SMS-based communication with patients via their smartphones upon arrival.
3	**Pain treatment quality review system**	18	**Leaflet's on pain management in the ED**
4	**Pain treatment in digital medical documentation**	19	**Teaching by experiencing—pain simulation workshops for ED staff**
5	**Posters with the process of pain treatment in the ED**	20	**Education of the Medical Rescue Team**
6	**Internal marketing**	21	**Expanded description of pain scale**
7	**Changes to the procedure for pain treatment**	22	**Awareness campaign: Throughout the area**
8	**Instruction: ED decision-making algorithms**	23	**Educational website, search engine positioning**
9	**Dispatcher: Suggestions on how to manage pain**	24	**General education campaign**
10	**Dispatcher: Decision-making algorithms for dispatchers**	25	**ED & Night Medical Care—redirection option**
11	**Dispatcher: checklist for pain treatment**	26	**Emergency Medical Services team educates the patient on self-medication**
12	**Dispatcher: Video Dispatcher Consultation**	27	**Pre-triage pain card**
13	**Dispatcher: Redirection to primary care facility**	28	**Ampullary at TRIAGE**
14	**Dispatcher: Redirect to Telephone Advice**	29	**ED staff can write drug administration orders**
15	**Feedback culture in ED Team**	30	**Conversation with the patient**
